# Hémorragie Cérébrale Post Envenimation par Morsure de Serpent Responsable D'une Cécité Irréversible Chez Un Enfant de 6 Ans au Mali

**DOI:** 10.48327/mtsibulletin.2021.116

**Published:** 2021-07-29

**Authors:** A. Yalcouyé, S.H. Diallo, S. Diallo, G. Landouré, T. Bagayoko, O. Maiga, Z. Fomba, D. Djibo, C.O. Guinto, Y. Maiga

**Affiliations:** 1 Faculté de médecine et d'odontostomatologie, USTTB, Bamako, Mali; 2 Service de neurologie, CHU Gabriel Touré, Bamako, Mali; 3 Service de neurologie, CHU Point G, Bamako, Mali; 4 Service d'anesthésie réanimation, CHU Gabriel Touré, Bamako, Mali

**Keywords:** Envenimation, Morsure de serpent, Cécité, Hémorragie cérébrale, Hôpital, Mali, Afrique subsaharienne, Envenomation, Snakebite, Blindness, Hemorrhagic stroke, Hospital, Mali, Sub-Saharan Africa

## Abstract

**Introduction:**

L'envenimation par morsure de serpent peut être à l'origine de graves séquelles. Nous rapportons le cas d'un enfant de sexe masculin de six ans, mordu par un serpent.

**Description clinique:**

L'enfant présentait une gingivorragie, une douleur abdominale, des vomissements sanguinolents et des céphalées intenses suite à une morsure de serpent. L'examen neurologique a retrouvé une paralysie du nerf III associée à une cécité bilatérale et une mydriase bilatérale, aréactive à droite. Le scanner cérébral a mis en évidence un hématome frontal gauche. L'évolution sous antivenin fut marquée par la disparition des signes cliniques, hormis la cécité toujours présente 18 mois après la sortie.

**Discussion - Conclusion:**

Le syndrome hémorragique évoquait une morsure de vipéridé. La cécité est rarement observée à la suite d'une envenimation vipérine. Dans notre cas, la présence de signes d'hypertension intracrânienne, l'absence de lésion oculaires et l'imagerie médicale sont en faveur d'une compression des nerfs optiques qui serait à l'origine de la cécité définitive.

## Abstract (en)

**Introduction:**

Snakebite envenomation can cause serious damage. Here, we report the case of a six-year-old male child bitten by a snake.

**Clinical description:**

The child presented a gingivorrhagia, abdominal pain, bloody vomiting and severe headache from a snakebite. Neurological examination showed paralysis of the III cranial nerve associated with bilateral blindness and mydriasis, unreactive on the right. The brain scan revealed a left frontal hematoma. The course on antivenom was marked by the disappearance of clinical signs except blindness which remained 18 months after discharge.

**Discussion - Conclusion:**

The hemorrhagic syndrome evoked viper bite. Blindness is rarely seen as a result of viperine envenomation. In our case, the presence of intracranial hypertension, absence of ocular lesions and scanner were in favor of compression of the optic nerves which resulted in permanent blindness.

## Introduction

Les morsures de serpent font partie des pathologies tropicales négligées dont les complications sont fréquentes et peuvent être dramatiques en milieu rural [5]. Elles sont estimées à environ 5,5 millions par an dans le monde, dont 1,8 million envenimations et 94000 décès [6].

Au Mali, la morbidité est estimée à 100 envenimations pour 100000 habitants par an [4]. Les manifestations de l'envenimation peuvent être locales et/ou générales, de gravité variable, dépendant notamment de l'espèce de serpent en cause. Au Mali, les vipéridés et les élapidés sont les familles les plus souvent impliquées dans les envenimations humaines. Les syndromes venimeux classiquement individualisés sont le syndrome vipérin et le syndrome cobraïque.

Le syndrome vipérin est marqué par une réaction inflammatoire loco-régionale, une hypotension artérielle et un syndrome hémorragique par coagulopathie de consommation [11]. L'envenimation peut provoquer des accidents vasculaires cérébraux ischémiques ou hémorragique [1]. La cécité, complication ophtalmologique grave, mais très peu rapportée en Afrique sub-saharienne, est le plus souvent liée à une toxicité directe du venin sur les yeux ou une lésion du lobe occipital [2, 10].

Nous rapportons le cas d'un enfant ayant développé une cécité bilatérale irréversible par envenimation dans le service de neurologie du CHU Gabriel Touré de Bamako.

## Observation Clinique

Il s'agit d'un garçon de 6 ans, issu d'une grossesse estimée à terme et d'un accouchement eutocique, ayant eu un développement psychomoteur normal, sans antécédents médicochirurgicaux particuliers connus. Il a été victime d'une morsure de serpent au niveau de la face dorsale du pied gauche, serpent dont l'espèce n'a pas été identifiée. La morsure est survenue au mois de juin en plein hivernage, alors que l'enfant se trouvait vers le crépuscule dans les broussailles à côté de son domicile dans un village. Les premiers symptômes consistaient en un oedème et une douleur locale au niveau du pied gauche apparus environ 12 heures après la morsure. Le lendemain, l'apparition d'une gingivorragie et d'une douleur abdominale ont conduit ses parents à l'amener au centre de santé de leur village. Il aurait bénéficié d'un traitement médical dont nous n'avons pas pu spécifier les molécules. Il a ensuite présenté des céphalées intenses et brutales le cinquième jour ce qui a motivé le transfert vers l'hôpital.

Il est admis au service d'accueil des urgences (SAU) le sixième jour après la morsure. On notait également une plaie au niveau du pied gauche au point de la morsure qu'un agent de santé rural aurait sucé au préalable afin, selon les croyances de la médecine traditionnelle, d'en « extraire les crochets du serpent », phénomène contredit par la médecine fondée sur les preuves. L'examen neurologique a mis en évidence un syndrome d'hypertension intracrânien (HIC) sur la présence de céphalées et d'une mydriase bilatérale aréactive à droite, d'un ptosis unilatéral à droite et d'une cécité bilatérale. L'examen réalisée par l'ophtalmologue avait aussi confirmé la cécité bilatérale.

Sur la numération formule sanguine, on notait une anémie à 8 g/dl normocytaire normochrome et une thrombopénie à 80000/mm^3^. Le résultat du test de coagulation sur tube sec réalisé à l'admission est revenu complètement incoagulable au premier test et partiellement coagulable au deuxième test réalisé 4 heures plus tard. Le taux de prothrombine, le temps de céphaline activée et la fibrinogénémie n'ont pas été dosés faute de moyen.

**Figure 1:**
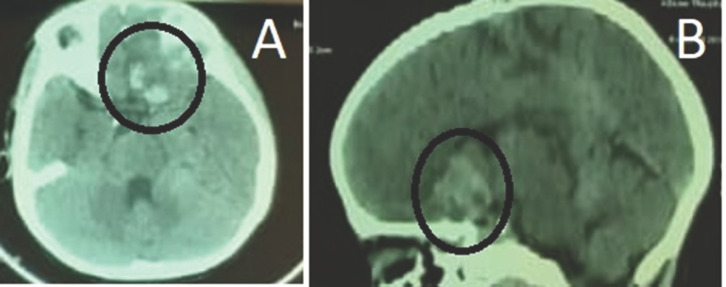
Scanner cérébral en coupe axiale (A) et sagittale (B) sans injection du produit de contraste mettant en évidence une hyperdensité spontanée frontale gauche entourée d'un oedème péri-lésionnel en faveur d'une hémorragie cérébrale intraparenchymateuse (cercle noir) Non contrast brain Computed Tomography scan in axial (A) and sagittal (B) cut, showing left frontal spontaneous hyperdensity with perilesional edema suggesting brain intraparenchymal haemorrhage (black circle)

L'échographie abdominopelvienne était normale. Le scanner cérébral en coupe axial et sagittal réalisé 7 jours après la morsure a mis en évidence un hématome intra-parenchymateux frontal gauche s'étendant dans la région traversée par les voies optiques (Fig. 1A et Fig. 1B).

Le traitement initial a associé du paracétamol par voie intraveineuse (60 mg/kg/jour), du mannitol 20% à raison de 5 ml/kg/jour en deux doses par jour et trois doses d'antivenin polyvalent (Inoserp^®^ Panafricain, Inosan Biopharma) par voie intraveineuse le sixième jour après l'envenimation. Il a ensuite bénéficié de trois poches de sang total iso-groupe et iso-rhésus.

L'évolution fut marquée par la disparition des signes cliniques et de l'hypertension intracrânienne, à l'exception de la persistance de la cécité bilatérale au 18e mois de suivi.

## Discussion

Les envenimations par morsure de serpent engendrent de nombreuses séquelles notamment neurologiques, rénales, fonctionnelles et plus rarement ophtalmologiques [2, 10]. Bien que l'espèce du serpent n'ait pas été identifiée, les signes présentés par le patient sont évocateurs d'une morsure par vipéridé et plus spécifiquement par *Echis ocellatus*. Dans une étude récente réalisée par Touré M et al, les espèces de serpent identifiées étaient fréquemment les vipéridés (*Echis* et *Bitis*) au Mali [9].

Des cas de cécité irréversible par atteinte des voies visuelles suite à des morsures de serpents ont été rapportés dans la littérature [7, 8], mais, à notre connaissance, c'est le premier cas rapporté au Mali. En effet, Katibi et al ont rapporté un cas de cécité chez un enfant de 10 ans survenue deux semaines après la morsure d'une vipère [9]. Chez ce patient, les auteurs ont décrit des lésions oculaires à type d'exophtalmie et de chémosis, plus tard compliqué d'opacité cornéenne bilatérale. Cependant, le cas de notre patient se distingue par la précocité de la survenue des symptômes qui était de quelques heures mais aussi l'absence de lésion oculaire objective à l'examen ophtalmologique. Cela montre à quel point les envenimations par morsure de vipéridés sont graves sur le plan fonctionnel et vital. L'hémorragie est classiquement vue dans le syndrome vipérin du fait de l'association de lésions vasculaires et d'une coagulopathie de consommation induites par le venin. Le mécanisme de survenue de la cécité se fait soit par atteinte directe des structures de l'oeil ou par atteinte des centres visuels occipitaux [8]. Dans le cas que nous rapportons, il n'y avait pas d'atteinte oculaire ni occipitale objectivable au scanner. La cécité serait probablement due à l'hypertension intracrânienne qui malgré sa prise en charge a entraîné une compression des nerfs optiques en raison de leur proximité avec la zone de saignement.

La prise en charge thérapeutique reste symptomatique. L'osmothérapie avec le mannitol permet de lutter contre l'oedème péri-lésionnel ce qui permet de diminuer l'hypertension intracrânienne. L'immunothérapie antivenimeuse, qui est un pilier dans la prise en charge de l'envenimation systémique, n'est malheureusement pas toujours disponible dans les centres de santé de référence et des hôpitaux, et encore moins dans les milieux ruraux au Mali [3, 8]. Le retard de prise en charge, pour des raisons diverses, comme l'éloignement du lieu de morsure ou le passage chez un tradipraticien, explique fréquemment les complications graves, notamment hémorragiques, qui surviennent chez ces patients et qui mettent en jeu le pronostic vital ou entraînent des séquelles fortement pénalisantes.

## Conclusion

L'envenimation par morsure de vipère peut être catastrophique si la prise en charge n'est pas rapide et adaptée. Les séquelles qui en découlent sont dramatiques. Bien que peu communément rapportés, des signes neurologiques s'associent souvent au tableau et devraient être recherchés devant toute envenimation par morsure de serpent. L'antivenin doit être disponible et accessible surtout à la population rurale qui est le plus souvent victime de ces envenimations. Il est nécessaire de définir un protocole de prise en charge des envenimations sévères, en les orientant le cas échéant vers un service spécialisé.

## Conflits D'intérêts

Les auteurs ne déclarent aucun conflit d'intérêt.
